# Cumulative Bisphenol A Release and Elution Kinetics from Pediatric Restorative and Orthodontic Resin-Based Materials: An In Vitro LC–MS/MS Investigation

**DOI:** 10.3390/children13060821

**Published:** 2026-06-16

**Authors:** Angelo Aliberti, Mirko Piscopo, Franklin Garcia-Godoy, Luigi Ausiello, Lucia Grumetto, Teresa Ponticorvo, Francesco Giordano

**Affiliations:** 1 Department of Neuroscience, Reproductive Science and Odontostomatological Sciences, University of Naples Federico II, Via Sergio Pansini, 5, 80138 Naples, Italy; mirk.piscopo@studenti.unina.it; 2 Department of Bioscience Research, College of Dentistry, University of Tennessee Health Science Center, Memphis, TN 38163, USA; godoy@uthsc.edu; 3 Department of Pharmacy, School of Medicine and Surgery, University of Naples Federico II, Via D. Montesano, 49, 80131 Naples, Italy; lu.ausiello@studenti.unina.it (L.A.); te.ponticorvo@studenti.unina.it (T.P.); 4 Department of Medicine, Surgery and Dentistry “Scuola Medica Salernitana”, University of Salerno, 84081 Baronissi, Italy; frgiordano@unisa.it

**Keywords:** Bisphenol-A (BPA), pediatric dentistry, LC-MS/MS, resin-based dental materials, artificial saliva, in vitro release, dental monomer, polymers, orthodontic materials

## Abstract

**Background**: Bisphenol A (BPA) release from resin-based dental materials is a growing concern due to its potential endocrine-disrupting effects, particularly in pediatric patients. This in vitro study evaluated cumulative BPA release and elution kinetics from commonly used pediatric resin-based materials, due to the limited evidence available. **Methods**: Three restorative materials (*Clearfil Majesty ES-2*, *Estelite Sigma Quick*, and *Stela Automix*) and one orthodontic material (*Transbond XT*) were investigated. Eighteen disk-shaped specimens (5.5 mm in diameter and 2 mm in thickness) were prepared for each material and immersed in artificial saliva (pH 6.8) at 37 °C for 1, 7, and 28 days. BPA concentrations were quantified using liquid chromatography–tandem mass spectrometry (LC–MS/MS). BPA release kinetics were evaluated during the early (1–7 days) and late (7–28 days) release phases. **Results**: All investigated materials released measurable BPA concentrations, with cumulative BPA release progressively increasing up to 28 days. *Clearfil Majesty ES-2* and *Estelite Sigma Quick* exhibited the highest cumulative BPA concentrations, whereas *Stela Automix* showed markedly lower values. *Transbond XT* also demonstrated measurable BPA release. For all materials, BPA release kinetics were significantly higher during the early phase than during the late phase (*p* < 0.001), indicating a non-linear release behavior over time. **Conclusions**: BPA release from pediatric restorative and orthodontic resin-based materials is material-dependent and characterized by progressive cumulative accumulation associated with significantly higher early-phase release rates. These findings highlight the importance of assessing the safety of resin-based materials used in pediatric dentistry.

## 1. Introduction

Resin-based restorative and orthodontic materials are widely used in pediatric dentistry because of their esthetic properties, adhesive capability, and minimally invasive clinical application. Resin composites are routinely employed for direct restorations in primary and permanent teeth, while resin-based orthodontic adhesives are extensively used for fixed orthodontic treatments in children and adolescents [1,2]. Despite their clinical advantages, increasing attention has been directed toward the potential release of residual monomers and degradation by-products from these materials into the oral environment [3,4,5,6].

Among the substances potentially released from dental resin-based materials, Bisphenol A (BPA) has emerged as one of the most debated compounds in contemporary dental biomaterials research [7]. BPA is a synthesized monomer used in the production of polycarbonate plastics and epoxy resins and structurally associated with several dental methacrylate monomers, i.e., bisphenol A glycidyl methacrylate (Bis-GMA) and bisphenol A ethoxylated dimethacrylate (Bis-EMA) [8,9]. Although BPA is not intentionally added to most contemporary restorative materials, trace amounts may still be present as impurities derived from manufacturing processes or may originate from hydrolytic and enzymatic degradation of BPA-derived monomers under oral conditions [10,11].

The growing concern regarding BPA exposure is primarily related to its recognized endocrine-disrupting activity. BPA can interact with estrogenic, androgenic, and thyroid hormone pathways and may alter several biological processes even at very low concentrations [12,13,14]. Experimental and epidemiological evidence has associated BPA exposure with oxidative stress, inflammatory dysregulation, altered neurodevelopment, metabolic disorders, and reproductive abnormalities [15,16,17,18,19]. Importantly, BPA does not appear to follow the traditional dose–response toxicological model, since biological effects have also been observed at low-dose chronic exposure levels [20].

Children and adolescents are considered particularly vulnerable to BPA exposure because of their lower body mass, immature detoxification systems, and ongoing endocrine, neurological, and immunological development [21,22]. Moreover, BPA exposure in pediatric patients is cumulative and may derive simultaneously from multiple environmental and dietary sources, including food packaging, beverages, toys, medical devices, and dental materials [23,24,25,26]. In pediatric dentistry, this issue becomes especially relevant because resin-based restorative and orthodontic materials may remain in the oral cavity for prolonged periods and are continuously exposed to saliva, temperature variations, masticatory loading, and enzymatic degradation [27,28].

In recent years, increasing toxicological concerns regarding BPA have also influenced international regulatory and clinical recommendations. The European Food Safety Authority (EFSA) drastically reduced the tolerable daily intake (TDI) of BPA to 0.2 ng/kg/day, highlighting the need to minimize chronic exposure, particularly in vulnerable populations [29]. Similarly, the recent WHO guideline on environmentally friendly and less invasive oral health care suggested limiting the use of resin-based fissure sealants and composites containing BPA derivatives in children, adolescents, and pregnant or breastfeeding women because of their increased susceptibility to potential endocrine effects [30]. These recommendations underline the growing international concern regarding BPA exposure from dental materials used in pediatric patients.

Modern resin-based materials used in pediatric dentistry differ considerably in monomer composition and polymer chemistry, factors that may directly influence BPA release behavior [31]. *Clearfil Majesty ES-2* (Kuraray) and *Estelite Sigma Quick* (Tokuyama Dental) are highly filled light-cured restorative composites containing Bis-GMA-based resin systems commonly used for pediatric restorations because of their favorable esthetics and handling characteristics [32]. In contrast, *Stela Automix* (SDI) is a self-cure restorative material developed to simplify restorative procedures and reduce chair time in pediatric patients through a simplified placement protocol [33]. Its formulation is mainly based on UDMA-derived chemistry and is marketed as free from conventional Bis-GMA monomers [34,35]. However, despite these compositional differences, limited evidence is currently available regarding their comparative BPA release behavior under simulated oral conditions.

Besides restorative composites, orthodontic adhesives and resin-based materials may represent an additional and often underestimated source of BPA exposure in pediatric patients [36]. *Transbond XT* (3M), one of the most used orthodontic bonding systems worldwide, contains Bis-GMA and TEGDMA monomers and may remain intraorally for several months or years during orthodontic treatment [37]. Under oral conditions, hydrolytic degradation, thermal fluctuations, and salivary enzymatic activity may progressively contribute to the release of residual monomers and BPA-related compounds over time [38,39,40]. Consequently, restorative and orthodontic materials may collectively contribute to chronic low-dose BPA exposure during critical developmental stages.

Previous studies showed that BPA release from resin-based dental materials is generally highest during the first hours or days after polymerization and tends to decrease over time [41,42,43]. Nevertheless, low-level release may persist for extended periods because of ongoing polymer degradation [38]. To date, most investigations have evaluated restorative materials or orthodontic adhesives and resin-based materials separately, while limited information is available regarding their comparative cumulative BPA release and release kinetics under identical experimental conditions, particularly for materials commonly employed in pediatric dentistry.

Simulated saliva systems are widely used in in vitro studies because they allow standardized simulation of oral conditions and controlled evaluation of material degradation and chemical elution over time [44,45]. Therefore, the aim of the present in vitro study was to evaluate the cumulative release and elution kinetics of Bisphenol A (BPA) from restorative and orthodontic resin-based materials commonly used in pediatric dentistry, including *Clearfil Majesty ES-2* (Kuraray), *Estelite Sigma Quick* (Tokuyama Dental), *Stela Automix* (SDI), and *Transbond XT* (3M), after immersion in artificial saliva (pH 6.8) at 37 °C for 1, 7, and 28 days. BPA release was quantified using liquid chromatography–tandem mass spectrometry (LC–MS/MS), a highly sensitive analytical method widely used for trace-level detection of compounds. The null hypothesis was that no significant differences in BPA cumulative release or release kinetics would be observed among the tested materials over time.

## 2. Materials and Methods

### 2.1. Specimen Preparation

Three restorative dental materials and one orthodontic material commonly used in pediatric dentistry were evaluated in the present study. The composition and main characteristics of the investigated materials are reported in Table 1.

Specimen fabrication was carried out in accordance with the manufacturers’ recommendations. *Stela Automix* (SDI) was prepared using a self-curing protocol, whereas *Transbond XT* (3M Unitek), *Estelite Sigma Quick* (Tokuyama), and *Clearfil Majesty ES-2* (Kuraray) were prepared using a light-curing procedure. No light-curing procedure was applied to *Stela Automix*. Based on previous studies, eighteen disks of each material (5.5 mm in diameter and 2 mm in thickness) were prepared, providing six independent specimens for each experimental time point and using custom-made Teflon molds to standardize the procedure and to ensure comparable exposed surface areas among the tested materials [41,46]. The approximate volume and total exposed surface area were 47.5 mm^3^ and 82.08 mm^2^, respectively, with a surface area-to-volume ratio of approximately 2.0 mm^−1^. Uniform specimen dimensions were adopted to maintain consistent exposed surface areas across all tested materials.

The light-curing materials (*Transbond XT*, *Estelite Sigma Quick* and *Clearfil Majesty ES 2*) were polymerized for 20 s using an LED light-curing unit (RADII-CAL CX Collimated LED Curing Light, SDI, Bayswater, Australia) equipped with a light guide tip with an internal diameter of 6.0 mm. The curing unit delivered an average irradiance of 1200 mW/cm^2^, which was verified using a calibrated LED radiometer (SDI, Victoria, Australia) before specimen fabrication. The light guide was positioned in contact with the glass plate covering the specimen surface (0 mm distance).

After curing, the samples were removed from the molds, and their surfaces were polished with a water-cooled rotary polisher (Ecomet 30, Buehler Ltd., Lake Bluff, IL, USA) using 800-grit sandpaper to remove the surface layer of resin that potentially contains unpolymerized residual monomers from the oxygen inhibition layer, as is commonly done in in vitro studies investigating monomer release from resin-based dental materials [10,27,41,42].

### 2.2. Chemicals, Standards, and Analytical Solutions

Following a previously described protocol [46], Bisphenol A (BPA, purity ≥ 99%) served as the reference standard for analytical validation and was obtained from Sigma-Aldrich (Milan, Italy). Methanol (MeOH, HPLC grade ≥ 99.9%) was sourced from the same supplier. In-house produced Milli-Q water showed a conductivity of 0.055 μS cm^−1^ at 25 °C (corresponding to a resistivity of 18.2 MΩ·cm). Additional ultrapure water was generated using an Elix Essential water purification system (Merck Millipore, Burlington, MA, USA). A stock solution of BPA was prepared by dissolving 2.0 ± 0.1 mg of the analyte in 5 mL of MeOH inside amber glass vials; this solution was kept at −20 °C for up to four months. Working standard solutions at concentrations of 0.1, 0.25, 0.5, 1, 2.5, 5.0, and 10.0 μg L^−1^ were obtained by mixing appropriate aliquots of the stock solution and diluting with MeOH. Before each use, the working solutions were allowed to equilibrate to room temperature and then vortexed for one minute.

### 2.3. BPA Cumulative Release Protocol and Release Kinetics Evaluation

Immediately after specimen preparation, each sample was transferred into an individual glass vial containing 2 mL of commercially available artificial saliva (SAGF). The artificial saliva was based on the Fusayama–Meyer formulation and adjusted to a pH of 6.8. For reproducibility purposes, all components and their concentrations are reported below: potassium chloride (KCl) 0.4 g/L, sodium chloride (NaCl) 0.4 g/L, calcium chloride dihydrate (CaCl_2_·2H_2_O) 0.906 g/L, monosodium phosphate dihydrate (NaH_2_PO_4_·2H_2_O) 0.69 g/L, sodium sulfide nonahydrate (Na_2_S·9H_2_O) 0.005 g/L, and urea 1.0 g/L [47].

Subsequently, according to protocols previously described in earlier investigations [46,48], the specimens were incubated at 37 °C for a fixed experimental interval of 1 day, 7 days, and 28 days (n = 6 specimens per material for each time point). At each scheduled interval (1, 7, and 28 days), 200 µL aliquots of artificial saliva were collected, transferred into glass vials equipped with inserts, and analyzed for BPA quantification using LC-ESI-QqQ-MS/MS. Since each specimen was not transferred into another vial, BPA concentrations were evaluated as cumulative release values over time. All specimens included in the present investigation were newly fabricated and analyzed specifically for this study, and no previously published experimental data were reused. This experimental design differs from our previously published BPA-release investigations, in which aliquots were collected with partial renewal of the storage medium at each sampling interval. Consequently, the BPA concentrations reported here represent cumulative accumulation within the same storage medium over time rather than interval-release measurements. In addition, the present protocol was specifically designed to evaluate BPA release kinetics and to directly compare restorative and orthodontic resin-based materials under identical experimental conditions. Although *Clearfil Majesty ES-2* and *Estelite Sigma Quick* were also investigated in previous studies, the data presented herein were generated from newly prepared specimens analyzed under the current experimental protocol [27,46].

To minimize potential BPA contamination, only glass laboratory materials were used during all experimental and analytical procedures, as plastic consumables may represent a potential source of BPA release. All glassware was thoroughly cleaned using organic solvents and ultrapure water before use. In addition, negative control samples consisting of artificial saliva without any restorative or orthodontic material were incubated and analyzed under identical experimental conditions to exclude possible BPA contamination originating from the storage medium, containers, environmental exposure, or analytical procedures.

To evaluate BPA release kinetics, the daily release rate was calculated for each replicate over two consecutive experimental intervals: from day 1 to day 7 (early release phase) and from day 7 to day 28 (late release phase). The daily release rate during the early phase was calculated as (C_7_ − C_1_)/6, whereas the late-phase release rate was calculated as (C_28_ − C_7_)/21, where C_1_, C_7_, and C_28_ correspond to the BPA concentrations (µg/L) measured at the respective time points.

### 2.4. LC–MS/MS Analytical Procedure for BPA Quantification

BPA quantification was performed using an analytical protocol based on a previously validated method, with minor adaptations for the present experimental design [46].

All analyses were carried out using an Agilent 6470 LC/ESI-TQ system (Agilent Technologies, Santa Clara, CA, USA) equipped with a Jet Stream ion source operating in negative ionization mode. Chromatographic separation was achieved using an Agilent 1290 Series UHPLC system (Santa Clara, CA, USA) coupled with a Luna Polar stainless-steel column (1.7 µm, 100 Å, 50 mm × 2.1 mm; Phenomenex, Torrance, CA, USA). The flow rate was maintained at 0.400 mL/min, the column temperature was set at 45 °C, and the injection volume was 5 µL.

The chromatographic separation was performed using a linear gradient consisting of 0.01% acetic acid in ultrapure water and 0.01% acetic acid in methanol (MeOH), as detailed in Table 2.

After each analytical run, the chromatographic column was re-equilibrated for 2 min. The mass spectrometer was periodically calibrated within a mass range of 112.99–2833.87 amu. Data acquisition and processing were performed using MassHunter Workstation software (version B.08.00, Agilent, Santa Clara, CA, USA). Analyses were conducted in multiple-reaction monitoring (MRM) mode. To optimize ion source parameters under LC flow conditions, standard BPA solutions at a concentration of 1000.0 ng/mL were injected. The optimized source conditions were as follows: gas temperature 200 °C, gas flow 11 L/min, nebulizer pressure 45 psi, sheath gas temperature 350 °C, sheath gas flow 12 L/min, ion spray voltage −3500 V, and nozzle voltage 2000 V.

MRM transitions were optimized by acquiring product ion spectra using the Optimizer software (version B.08.00) supplied by the instrument manufacturer (Agilent, Santa Clara, CA, USA). MS/MS analyses were performed in negative multiple-reaction monitoring mode (−MRM). For each precursor ion (Q1), two product ions (Q3) were selected: one transition was selected for quantification (quantifier ion, Q), whereas the second transition was used as a qualifier ion (q) for confirmation purposes. Both Q1 and Q3 operated at unit resolution, using a cell accelerator voltage of 7 V and a dwell time of 150 ms for each monitored transition. BPA identification was based on comparing the retention time (tR) of the chromatographic peaks of both the quantifier and qualifier ions with those of reference standards. The specific UHPLC-MS/MS transitions used for BPA are listed in Table 3.

### 2.5. Analytical Sensitivity and Quantification Limits

The limit of detection (LOD) and limit of quantification (LOQ) were determined based on signal-to-noise ratio criteria. Specifically, the LOD was set as the analyte concentration yielding a signal-to-noise ratio of 3, while the LOQ was defined at a ratio of 10. Under the experimental conditions applied here, the LOD and LOQ for BPA were approximately 0.03 µg/L and 0.10 µg/L, respectively. These figures represent the lowest BPA amounts that could be reliably detected and quantified, thereby confirming that the analytical method is suitable for trace-level measurement of BPA.

Any concentration falling below the LOQ was recorded as “<LOQ” and omitted from quantitative statistical analyses, because values under the quantification limit cannot be expressed as precise numerical concentrations with confidence. For materials where all replicate measurements remained below the LOQ, the results were only described qualitatively and were not included in the quantitative statistical evaluation.

This practice aligns with standard conventions in analytical chemistry for situations where all measurements in each experimental group are below the quantification limit, since substituting numerical values would introduce artificial data not directly supported by the analytical measurements.

### 2.6. Statistical Analysis

Sample size calculation was performed through an a priori power analysis using G*Power software (version 3.1.9.7, Heinrich Heine University Düsseldorf, Düsseldorf, NRW, Germany). The experimental design included two independent factors: material type (four levels: *Stela Automix*, *Transbond XT*, *Estelite Sigma Quick*, and *Clearfil Majesty ES-2*) and exposure time (three levels: 1, 7, and 28 days). A two-way ANOVA model was assumed for the analysis. Since no preliminary data were available for the investigated materials, a medium effect size (f = 0.25) was conservatively selected according to Cohen’s criteria. The significance level was set at α = 0.05 with a target statistical power of 0.80. The minimum total sample size required was 72 experimental units, corresponding to six independent replicates for each combination of material and time points (4 materials × 3 time points × 6 replicates = 72). Throughout the experimental procedure, temperature and pH were maintained constant at 37 °C and 6.8, respectively.

Statistical analyses were performed using STATA software (version 14.0; StataCorp LLC, College Station, TX, USA). A two-way analysis of variance (ANOVA) was used to evaluate the effects of material type and exposure time on BPA concentration values. The dependent variable was BPA concentration (µg/L), whereas material type, observation time, and their two-way interaction were included as fixed factors in the model. Model assumptions were assessed through residual analysis. Residual normality was evaluated using the Shapiro–Wilk test. The significance of the main effects and their interaction was determined by two-way ANOVA. When significant effects or interactions were identified, pairwise post hoc comparisons were performed using Tukey’s test.

For the evaluation of BPA release kinetics, paired *t*-tests were performed separately for each material to compare the early (from day 1 to day 7) and late (from day 7 to day 28) release rates. The analysis was conducted using the six paired release-rate values obtained from the six independent replicates for each material. A statistically significant result indicated a variation in release velocity between the two experimental intervals.

Descriptive statistics, including mean, standard deviation, minimum, and maximum values, were calculated for each material and time point. Statistical significance was established at *p* < 0.05 for all analyses.

## 3. Results

### 3.1. BPA Cumulative Release

Bisphenol A (BPA) quantification was performed for each of the four investigated dental materials after immersion periods of 1, 7, and 28 days. Throughout the experimental period, temperature and pH were maintained constant at 37 °C and 6.8, respectively. Six independent specimens were analyzed for each combination of material and exposure time.

**Figure 1:**
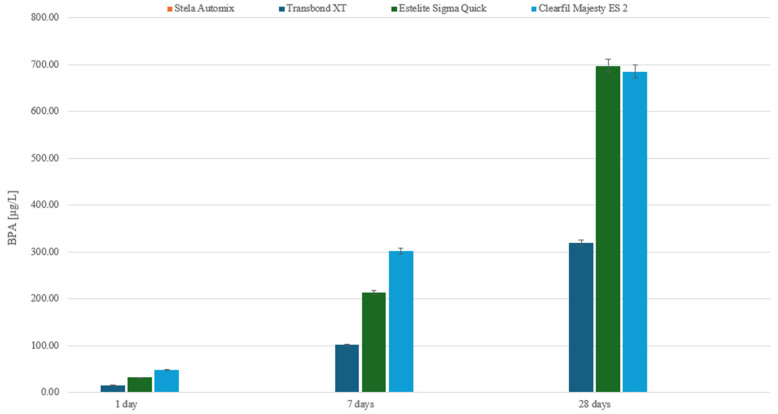
Comparison of cumulative BPA release from all tested materials in artificial saliva (pH 6.8) at 37 °C and three observation times (1, 7 and 28 days).

Descriptive statistical data, including mean BPA concentrations and corresponding standard deviations expressed in µg/L, are reported in Table 4 and graphically illustrated in Figure 1.

Since the storage medium was not replaced during the experimental period, the measured BPA concentrations represented cumulative release values from day 1 to day 28.

**Figure 2:**
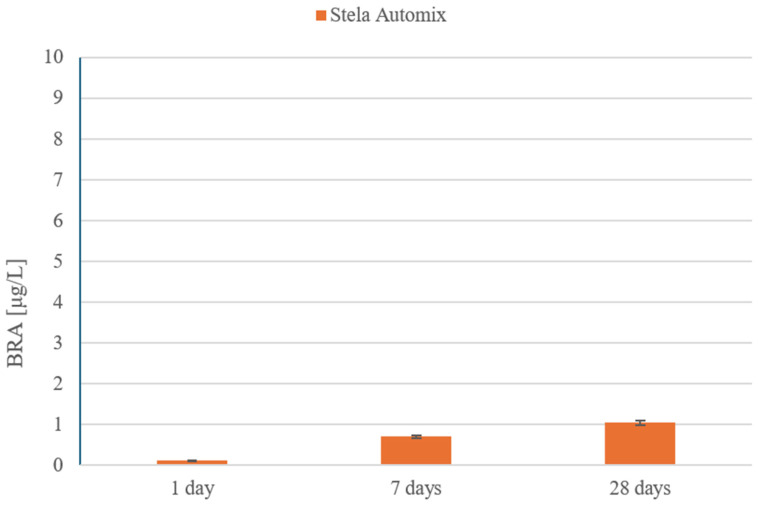
Temporal profile of cumulative BPA release in *Stela Automix* at pH = 6.8, 37 °C and at three different observation times (1, 7 and 28 days).

Regarding *Stela Automix*, the mean BPA concentration measured after 1 day of immersion was 0.118 ± 0.008 μg/L. As shown in Figure 2, the concentration increased to 0.708 ± 0.015 μg/L after 7 days and further rose to 1.045 ± 0.019 μg/L after 28 days of immersion.

**Figure 3:**
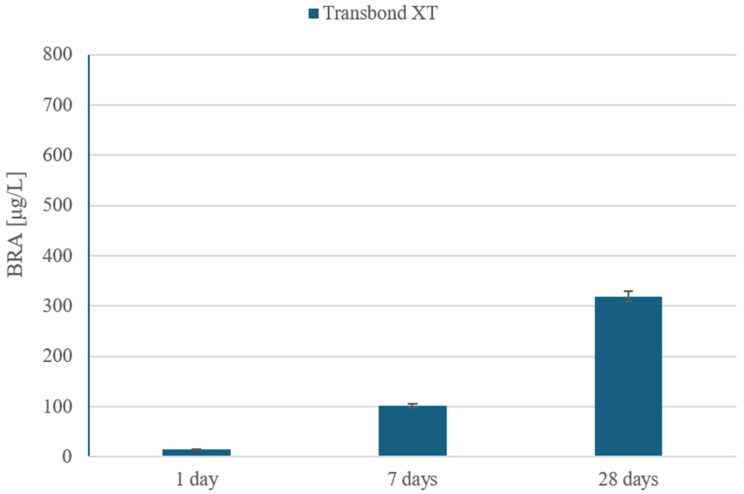
Temporal profile of cumulative BPA release in *Transbond XT* at pH = 6.8, 37 °C and at three different observation times (1, 7 and 28 days).

For *Transbond XT*, the mean BPA concentration was 15.45 ± 0.18 μg/L after 1 day of immersion. As shown in Figure 3, the concentration progressively increased to 101.93 ± 1.01 μg/L after 7 days and reached 319.33 ± 2.46 μg/L after 28 days.

**Figure 4:**
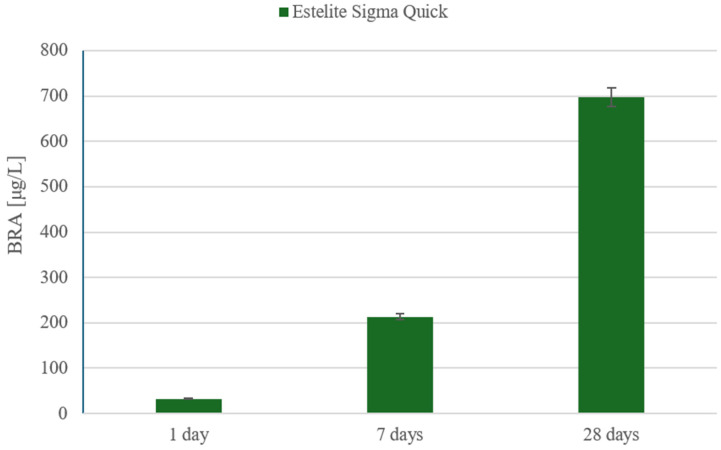
Temporal profile of cumulative BPA release in *Estelite Sigma Quick* at pH = 6.8, 37 °C and at three different observation times (1, 7 and 28 days).

As for *Estelite Sigma Quick*, the mean BPA concentration was 32.03 ± 0.39 μg/L after 1 day of immersion. As illustrated in Figure 4, BPA release increased to 213.0 ± 1.9 μg/L after 7 days and further rose to 697.6 ± 5.6 μg/L after 28 days.

**Figure 5:**
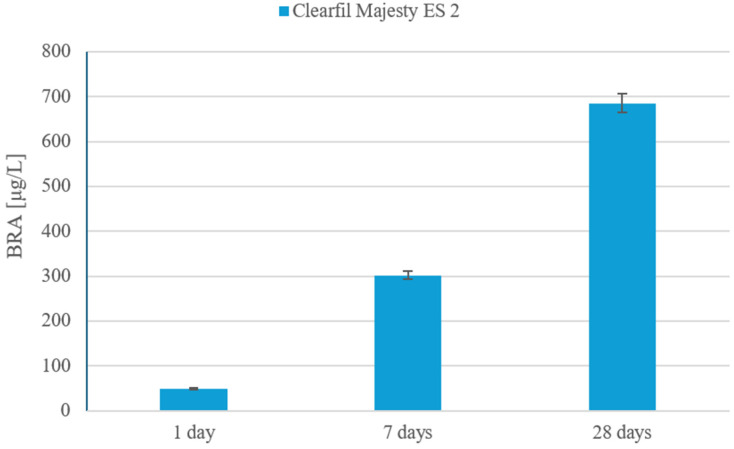
Temporal profile of cumulative BPA release in *Clearfil Majesty ES 2* at pH = 6.8, 37 °C and at three different observation times (1, 7 and 28 days).

Finally, *Clearfil Majesty ES-2* showed a mean BPA concentration of 48.53 ± 0.60 μg/L after 1 day of immersion. As shown in Figure 5, BPA release increased to 301.72 ± 2.54 μg/L after 7 days and reached 685.47 ± 5.23 μg/L at the final experimental time point of 28 days.

Two-way ANOVA demonstrated significant main effects for both material type (*p* < 0.0001) and exposure time (*p* < 0.0001), as well as a significant material × time interaction (*p* < 0.0001). Residual analysis confirmed that model assumptions were satisfied, with residuals showing normal distribution according to the Shapiro–Wilk test (*p* = 0.18).

Due to the significant interaction, post hoc comparisons (Tukey’s test) were performed separately for each time point. At all three time points, all pairwise comparisons between materials were statistically significant (*p* < 0.001). Notably, at day 28, the difference between *Estelite Sigma Quick* and *Clearfil Majesty ES-2*, while still significant, had a higher *p*-value (*p* = 0.017) compared to the other comparisons.

Within each material group, all pairwise comparisons among the different time points revealed statistically significant increases in BPA concentration over time (*p* < 0.001).

### 3.2. BPA Release Kinetics

The kinetics of BPA release were further evaluated to determine whether BPA release occurred more rapidly during the initial phase of immersion compared with the subsequent experimental period. For all investigated materials, the mean daily BPA release rate was consistently higher during the early phase (1–7 days) than during the late phase (7–28 days).

Specifically, *Stela Automix* showed a decrease in the daily release rate from 0.098 to 0.016 μg/L/day. Similarly, *Transbond XT* exhibited a reduction from 14.41 to 10.35 μg/L/day, whereas *Estelite Sigma Quick* decreased from 30.16 to 23.08 μg/L/day. *Clearfil Majesty ES-2* also showed a marked reduction in daily BPA release, decreasing from 42.20 to 18.27 μg/L/day.

Paired *t*-tests performed on individual replicate values demonstrated statistically significant differences between the early and late release phases for all investigated materials (*p* < 0.001 for all comparisons).

**Figure 6:**
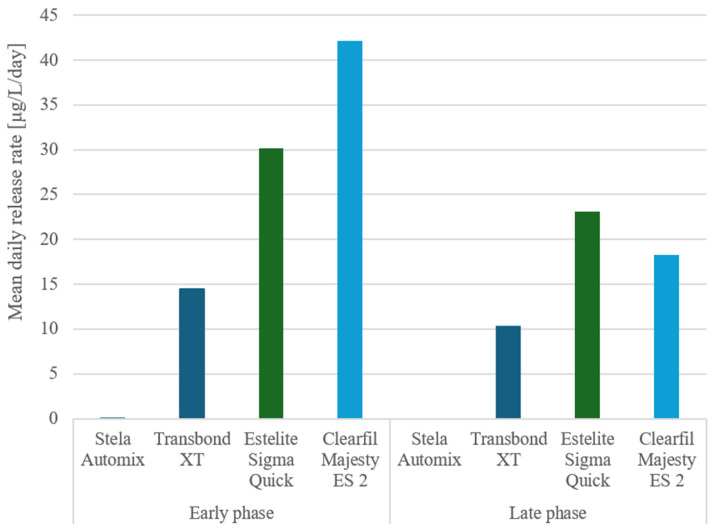
Comparison of mean daily BPA release rates (μg/L/day) between the early (1–7 days) and late (7–28 days) release phases for the investigated restorative and orthodontic resin-based materials.

These findings indicate that BPA release did not follow a linear pattern over time. The highest release rates were observed during the first 7 days of immersion, followed by a progressive reduction in the daily release rate during the subsequent experimental period. All release kinetics data are summarized in Table 5 and graphically illustrated in Figure 6.

## 4. Discussion

The present in vitro investigation evaluated both the cumulative release and the release kinetics of Bisphenol A from restorative and orthodontic resin-based materials commonly used in pediatric dentistry after immersion in artificial saliva at 37 °C. The results demonstrated that all investigated materials released measurable amounts of BPA over time, although with substantial differences depending on material composition and resin chemistry. More specifically, cumulative BPA concentrations progressively increased at each experimental time point up to 28 days for all tested materials. However, despite this continuous cumulative increase, the kinetic analysis demonstrated that BPA release occurred significantly more rapidly during the early phase (1–7 days) than during the late phase (7–28 days). Therefore, cumulative BPA accumulation and BPA release kinetics should be interpreted as two distinct but closely related processes.

This distinction represents one of the most important findings of the present study. While BPA concentrations continuously accumulated within the artificial saliva throughout the 28-day observation period, the daily amount released by the materials progressively decreased after the first week of immersion. In practical terms, this means that BPA continued to accumulate over time, although the release velocity became progressively lower during the later stages of the experiment. These findings strongly suggest that BPA release from resin-based dental materials does not follow a linear pattern, but rather a biphasic kinetic behavior characterized by an initial burst-release phase followed by slower long-term diffusion and degradation phenomena.

The significantly higher release kinetics observed during the early phase may reasonably be explained by the rapid diffusion of residual unreacted monomers and superficial low-molecular-weight compounds located within the oxygen inhibition layer and outer polymer network immediately after polymerization [3,5,49,50]. Following this initial diffusion process, BPA release progressively becomes dependent on slower hydrolytic degradation mechanisms occurring within the deeper polymer matrix [51,52,53]. Water sorption, hydrolysis of ester bonds, salivary penetration, and gradual polymer degradation may therefore contribute to prolonged low-level BPA release over time [42]. Similar degradation and diffusion mechanisms have previously been described for resin-based restorative materials and methacrylate-derived monomers [54,55].

From a material-dependent perspective, *Clearfil Majesty ES-2* and *Estelite Sigma Quick* showed the highest cumulative BPA concentrations among the tested restorative materials. Both materials contain Bis-GMA-based resin systems, which are widely recognized as potential sources of BPA-related compounds because residual BPA traces may derive either from impurities generated during monomer synthesis or from degradation of BPA-derived monomers under aqueous conditions [10,41,56]. Interestingly, although both composites demonstrated elevated cumulative release, their kinetic behavior showed slight differences over time. *Clearfil Majesty ES-2* exhibited the highest early release rate, whereas *Estelite Sigma Quick* showed the highest cumulative BPA concentration after 28 days. This finding suggests that the initial superficial diffusion process and the subsequent long-term degradation behavior may depend on different physicochemical characteristics of the restorative material, including polymer cross-link density, degree of conversion, filler content, water sorption behavior, and polymer network stability.

The present results are consistent with previous investigations evaluating BPA release from contemporary restorative composites [37,46]. In a recent time-dependent in vitro study conducted by De Nys et al., modern resin-based restorative materials also demonstrated progressive BPA accumulation over time, with higher release occurring during the initial experimental phase [41]. Similarly, previous studies reported that Bis-GMA-containing restorative systems generally exhibit greater BPA-related elution compared with alternative monomer formulations [38,39,41,43]. Taken together, these observations reinforce the hypothesis that resin composition remains one of the principal determinants of BPA release behavior.

*Transbond XT* also demonstrated measurable cumulative BPA release and elevated early-phase release kinetics. This finding is particularly relevant in pediatric dentistry because orthodontic resin-based materials may remain intraorally for prolonged periods during fixed orthodontic treatment, frequently for several months or years [57,58,59,60]. Consequently, unlike direct restorative procedures, orthodontic materials may contribute to chronic low-dose BPA exposure during critical developmental phases. Previous investigations already suggested that orthodontic adhesives can release BPA and estrogen-like compounds under simulated oral conditions [61,62]. However, the present investigation additionally demonstrated that orthodontic resin-based materials also follow a characteristic kinetic profile, with markedly greater release during the first week after immersion. Clinically, this finding may be especially relevant immediately after bracket bonding procedures, when salivary exposure to residual monomers is likely to be highest.

Conversely, *Stela Automix* exhibited substantially lower cumulative BPA concentrations and lower release kinetics throughout the entire observation period. This behavior may reasonably be attributed to its predominantly UDMA-based formulation and the absence of conventional Bis-GMA monomers. UDMA-based resin systems have increasingly attracted attention as potential alternatives to traditional Bis-GMA-containing materials to reduce BPA-related exposure [63,64]. Nevertheless, despite being marketed as a BPA-free restorative material, *Stela Automix* still exhibited measurable BPA release, although at significantly lower levels compared with the other tested materials. This observation is particularly important because it confirms that materials labeled as “BPA-free” cannot automatically be considered completely free from BPA release. Trace BPA concentrations may still originate from manufacturing impurities, degradation pathways, or minor undeclared resin components, as previously suggested in recent analytical investigations [27,43,45,46].

From a pediatric toxicological perspective, the present findings deserve particular attention. BPA is currently recognized as an endocrine-disrupting compound capable of interacting with estrogenic, androgenic, and thyroid signaling pathways even at very low concentrations [65,66,67]. Importantly, BPA biological activity does not always follow the traditional dose-dependent toxicological model, since chronic low-dose exposure may still induce biologically relevant effects [20]. Children and adolescents represent particularly vulnerable populations because of their lower body mass, immature detoxification systems, and ongoing endocrine and neurological development [68,69]. Moreover, BPA exposure in pediatric patients is cumulative and derives simultaneously from multiple environmental and dietary sources, including food packaging, beverages, toys, thermal papers, medical devices, dental materials and treatments [24,70,71,72]. Although measurable BPA concentrations were detected in all investigated materials, the practical significance of these findings should be interpreted with caution. The concentrations measured in the present study cannot be directly translated into systemic exposure levels because the experimental model did not reproduce oral clearance, swallowing, absorption, distribution, metabolism, or excretion processes. Nevertheless, the detection of BPA from all tested materials is consistent with previous investigations reporting BPA release from resin-based dental materials and supports ongoing concerns regarding cumulative BPA exposure from multiple environmental and dietary sources. Therefore, while the present findings confirm that resin-based materials may represent a source of BPA release, the actual toxicological relevance of the detected concentrations remains to be established through appropriately designed biological and clinical studies.

In this context, these findings become particularly relevant when considered considering the increasing international concern regarding chronic BPA exposure in vulnerable populations. Regulatory agencies and health care organizations have progressively emphasized the importance of reducing avoidable BPA exposure, especially during critical developmental stages. In particular, the recent reduction in the tolerable daily BPA intake proposed by the European Food Safety Authority (EFSA) and the recommendations issued by the World Health Organization regarding the cautious use of BPA-derived resin materials in pediatric patients further support the clinical relevance of minimizing unnecessary BPA exposure whenever possible [29,30]. Although the BPA concentrations measured in the present investigation cannot be directly translated into systemic exposure values, the progressive cumulative increase observed up to 28 days suggests that restorative and orthodontic materials may represent a potential source of BPA exposure. However, this interpretation should be made with caution because the present experimental model was conducted under static laboratory conditions and did not reproduce important clinical factors such as salivary flow, enzymatic activity, thermal fluctuations, biofilm formation, and mechanical loading, all of which may influence BPA release behavior in vivo. Furthermore, the novelty of the present study is the methodological approach adopted for cumulative BPA evaluation. Unlike many previous investigations in which the storage medium was totally replaced at each sampling, the artificial saliva solution used in the present protocol was intentionally maintained throughout the entire experimental period. Consequently, the measured BPA concentrations represented cumulative accumulation over time rather than isolated interval release values. This methodological design probably provides a more realistic simulation of progressive intraoral BPA accumulation during prolonged material exposure. At the same time, the kinetic analysis allowed differentiation between cumulative concentration increase and actual daily release velocity. Therefore, the combination of cumulative release assessment and release kinetics analysis represents one of the principal original aspects of the present investigation.

These findings should also be interpreted within the broader context of restorative material degradation and physicochemical stability. Previous studies demonstrated that polymerization shrinkage stress, hydrolytic degradation, water sorption, and interfacial instability may significantly influence the long-term behavior of resin-based restorative systems [73,74,75]. Finite element analyses evaluating polymeric restorative materials further demonstrated that stress distribution and polymer network behavior vary considerably depending on resin composition and filler characteristics [76,77,78]. Similarly, recent laboratory investigations evaluating ion-releasing restorative materials demonstrated that resin composition strongly affects chemical release dynamics under simulated oral conditions [79,80]. Overall, these findings support the concept that BPA release behavior cannot be interpreted independently from the broader physicochemical characteristics of restorative polymers.

A further clinically relevant consideration concerns the possibility of minimizing BPA exposure through appropriate operative strategies. Previous investigations demonstrated that the degree of polymerization and the integrity of the superficial resin layer may significantly influence residual monomer release [81,82]. Consequently, careful light-curing procedures, adequate curing times, finishing and polishing protocols, and removal of the oxygen inhibition layer may contribute to reducing the initial burst release of BPA-related compounds. In pediatric dentistry, where repeated restorative and orthodontic procedures may occur throughout childhood and adolescence, clinicians should carefully balance the mechanical and esthetic advantages of resin-based materials with their potential for chemical release. Whenever clinically feasible, the selection of restorative systems characterized by lower BPA release profiles may represent a prudent preventive strategy for vulnerable pediatric patients.

The employment of LC–MS/MS to detect even low concentration levels of BPA allows highly sensitive and selective trace-level quantification of endocrine-disrupting compounds compared to other detection techniques, minimizing analytical interference [83]. This methodological sensitivity is particularly important when evaluating BPA because biologically relevant effects may occur even at extremely low concentrations.

Despite the relevance of our findings, some limitations should be acknowledged. First, this was an in vitro study conducted under standardized laboratory conditions. The oral environment is considerably more complex and dynamic, involving salivary enzymes, bacterial biofilm, thermal fluctuations, dietary acids, pH changes, and mechanical loading, all of which may influence long-term degradation and BPA release behavior. In addition, the standardized disk-shaped specimens used in the present study allowed direct comparison among materials but did not reproduce the complex geometry, surface roughness, marginal configuration, or clinical thickness of actual restorations and orthodontic bonding applications. Moreover, although the cumulative model without medium replacement was useful for assessing progressive BPA accumulation, it does not fully reproduce the continuous salivary flow and clearance occurring in vivo, which may influence BPA dilution, removal, and local exposure dynamics. Therefore, caution should be exercised when extrapolating the present results directly to clinical conditions.

Second, the observation period was limited to 28 days and therefore reflects short- to medium-term release behavior rather than long-term clinical aging. Since restorative and orthodontic materials may remain intraorally for several years, future studies should investigate longer experimental periods under more clinically realistic conditions, including thermocycling, enzymatic degradation, mechanical fatigue, and biofilm exposure models. Third, only BPA release was evaluated in the present investigation. Other residual monomers and degradation by-products, including TEGDMA, UDMA, Bis-EMA-related compounds, and Bis-GMA derivatives, may also contribute to the biological effects associated with resin degradation and should therefore be included in future multi-analyte investigations. Furthermore, the present study was designed as an analytical investigation of BPA release and did not include any biological, cytotoxic, or toxicological assessment of the collected eluates. Therefore, the biological significance of the detected BPA concentrations cannot be directly inferred from the present findings. Future studies should combine chemical release analyses with appropriate biological models to better clarify the potential clinical implications of BPA exposure associated with resin-based dental materials.

Future in vivo studies evaluating salivary and urinary BPA concentrations after placement of pediatric restorative and orthodontic materials would provide clinically relevant information regarding the actual contribution of dental materials to systemic BPA exposure. Furthermore, additional research should focus on the development and validation of alternative resin systems characterized by lower endocrine-disrupting potential while maintaining adequate mechanical, adhesive, and esthetic properties. Taken together, these findings further support the need for continued research aimed at optimizing the physicochemical and biological performance of resin-based materials while minimizing potential endocrine-disrupting exposure in pediatric patients.

## 5. Conclusions

Despite the limitations inherent to this in vitro investigation, all tested restorative and orthodontic resin-based materials released measurable BPA concentrations under simulated oral conditions. Cumulative BPA release progressively increased throughout the 28-day experimental period for all investigated materials, whereas BPA release kinetics were significantly greater during the early release phase (1–7 days) compared with the late phase (7–28 days), demonstrating a non-linear release behavior over time. BPA release was strongly material-dependent, with Bis-GMA-based restorative materials exhibiting the highest cumulative concentrations and release rates, while the predominantly UDMA-based material demonstrated substantially lower BPA release. In addition, the orthodontic resin-based material evaluated in the present study showed measurable BPA release, highlighting the importance of considering potential long-term exposure associated with orthodontic treatments in pediatric patients.

The present findings emphasize the importance of evaluating both cumulative BPA release and release kinetics when characterizing the chemical release profile of resin-based materials used in pediatric dentistry. Furthermore, these results support the need for continued development and clinical validation of restorative and orthodontic materials aimed at reducing exposure to endocrine-disrupting compounds. Further biological and toxicological investigations are required to determine the clinical significance of the BPA concentrations detected in the present study.

## Figures and Tables

**Table 1:** Tested materials specifications (material information adapted from previous investigations [46]).

Material	Manufacturer	Type	Curing Mechanism	Composition
**Stela Automix** (LT n.1243437)	SDI (Victoria, Australia)	Resin-based restorative material	Self-curing	10–25% UDMA, 5–15% GDMA, 1–10% silica amorphous, 3–7% ytterbium fluoride, 1–5% MDP
**Transbond XT** (LT n. 10783797)	3M, Unitek (Monrovia, CA, USA)	Orthodontic resin-based material	Light-curing	70–80% Silane Treated Quartz, 10–20% BIS-GMA, 5–10% BIS-EMA, <2% Silane Treated Silica, <0.2% Diphenyliodonium Hexafluorophosphate
**Estelite Sigma Quick** (LT n.350M45)	Tokuyama Dental (Tokyo, Japan)	Resin composite	Light-curing	Bis-GMA, TEGDMA, Composite filler, Silica-zirconia filler, Photo initiator
**Clearfil Majesty ES-2** (LT n.5F0093)	Kuraray Co., Ltd. (Osaka, Japan)	Resin composite	Light-curing	Bis-GMA, Hydrophobic aliphatic dimethacrylate, Silanated barium glass filler, Organic filler/78 wt% Silanated barium glass filler: 0.7 μm, Organic filler: <100 μm

UDMA: urethane dimethacrylate; GDMA: glycidyl dimethacrylate; MDP: 10-methacryloyloxydecyl dihydrogen phosphate; Bis-GMA: bisphenol-glycidyl methacrylate; Bis-EMA: 2,2-bis(4-(2-methacryloxyethoxy) phenyl) propane; TEGDMA: triethylene glycol dimethacrylate.

**Table 2:** Gradient chromatographic elution protocol optimized for BPA separation (adapted from [46]).

Time (min)	Ultrapure Water with 0.01% Acetic Acid (%)	MeOH with 0.01% Acetic Acid (%)
0.0	60.0	40.0
0.5	60.0	40.0
3.0	5.0	95.0
4.0	5.0	95.0

**Table 3:** UHPLC–MS/MS quantifier and qualifier transitions used for BPA determination (adapted from [46]).

Ion	Transition (Q1 → Q3)	Collision Energy (eV)	Fragmentor (V)	Function
BPA-Q	227.2 → 133.0	−20	162	Quantifier
BPA-q	227.2 → 211.8	−28	162	Qualifier

**Table 4:** Cumulative BPA concentrations (µg/L ± SD) released from the tested materials after immersion in artificial saliva (pH 6.8) at 37 °C for 1, 7, and 28 days.

Material	BPA Release (μg/L ± SD)		
	1 Day	7 Days	28 Days
**Stela Automix** (LT n.1243437)	0.118 ± 0.008	0.708 ± 0.015	1.045 ± 0.019
**Transbond XT** (LT n. 10783797)	15.45 ± 0.18	101.93 ± 1.01	319.33 ± 2.46
**Estelite Sigma Quick** (LT n.350M45)	32.03 ± 0.39	213.0 ± 1.9	697.6 ± 5.6
**Clearfil Majesty ES 2** (LT n.5F0093)	48.53 ± 0.60	301.72 ± 2.54	685.47 ± 5.23

**Table 5:** Daily BPA release rates (μg/L/day) calculated for the early (1–7 days) and late (7–28 days) release phases of the investigated restorative and orthodontic resin-based materials.

Phase	Materials	Mean Release Rate (μg/L/day)
Early phase (from day 1 to day 7)	Stela Automix	0.098
	Transbond XT	14.41
	Estelite Sigma Quick	30.16
	Clearfil Majesty ES 2	42.20
Late phase (from day 7 to day 28)	Stela Automix	0.016
	Transbond XT	10.35
	Estelite Sigma Quick	23.08
	Clearfil Majesty ES 2	18.27

## Data Availability

The original contributions presented in this study are included in the article. Further inquiries can be directed to the corresponding authors.
